# Cloning and Functional Characterization of a Vacuolar Na^+^/H^+^ Antiporter Gene from Mungbean (*VrNHX1*) and Its Ectopic Expression Enhanced Salt Tolerance in *Arabidopsis thaliana*

**DOI:** 10.1371/journal.pone.0106678

**Published:** 2014-10-28

**Authors:** Sagarika Mishra, Hemasundar Alavilli, Byeong-ha Lee, Sanjib Kumar Panda, Lingaraj Sahoo

**Affiliations:** 1 Department of Biotechnology, Indian Institute of Technology Guwahati, Guwahati, India; 2 Department of Life Science, Sogang University, Mapo-gu, Seoul, Korea; 3 Department of Life Sciences and Bioinformatics, Assam University, Silchar, India; 4 Department of Biochemistry & Molecular Biology, Noble Research Centre, Oklahoma State University, Stillwater, OK, United States of America; Institute of Crop Sciences, China

## Abstract

Plant vacuolar NHX exchangers play a significant role in adaption to salt stress by compartmentalizing excess cytosolic Na^+^ into vacuoles and maintaining cellular homeostasis and ionic equilibrium. We cloned an orthologue of the vacuolar Na^+^/H^+^ antiporter gene, *VrNHX1* from mungbean (*Vigna radiata*), an important Asiatic grain legume. The *VrNHX1* (Genbank Accession number JN656211.1) contains 2095 nucleotides with an open reading frame of 1629 nucleotides encoding a predicted protein of 542 amino acids with a deduced molecular mass of 59.6 kDa. The consensus amiloride binding motif (^84^LFFIYLLPPI^93^) was observed in the third putative transmembrane domain of *VrNHX1*. Bioinformatic and phylogenetic analysis clearly suggested that *VrNHX1* had high similarity to those of orthologs belonging to Class-I clade of plant NHX exchangers in leguminous crops. *VrNHX1* could be strongly induced by salt stress in mungbean as the expression in roots significantly increased in presence of 200 mM NaCl with concomitant accumulation of total [Na^+^]. Induction of *VrNHX1* was also observed under cold and dehydration stress, indicating a possible cross talk between various abiotic stresses. Heterologous expression in salt sensitive yeast mutant AXT3 complemented for the loss of yeast vacuolar *NHX1* under NaCl, KCl and LiCl stress indicating that *VrNHX1* was the orthologue of *ScNHX1*. Further, AXT3 cells expressing *VrNHX1* survived under low pH environment and displayed vacuolar alkalinization analyzed using pH sensitive fluorescent dye BCECF-AM. The constitutive and stress inducible expression of *VrNHX1* resulted in enhanced salt tolerance in transgenic *Arabidopsis thaliana* lines. Our work suggested that *VrNHX1* was a salt tolerance determinant in mungbean.

## Introduction

Soil salinity poses increasing threat to plant growth and agricultural productivity worldwide [1]. More than 20% of the cultivated area and nearly half of the world's irrigated lands are adversely affected by salinity [2]. Enhanced crop production on salinity inflicted areas will rely on innovative agronomic practices coupled with use of genetically improved crop varieties [3]. In saline soils, Na^+^ is the predominant toxic ion. Excess accumulation of Na^+^ in cytosol is detrimental to many metabolic and physiological processes, vital for plant growth and productivity, as it causes ion imbalance, hyper osmotic stress, and oxidative damage to plants [4]. To cope with salinity stress, plants have evolved sophisticated mechanisms, including restricted uptake/exclusion of Na^+^ from cell, and compartmentalization of Na^+^ into vacuoles. Na^+^ efflux is catalyzed by a plasma membrane Na^+^/H^+^ antiporter (NHX) encoded by *SOS1* [5], [6] while, a vacuolar Na^+^/H^+^ antiporter catalyzes the sequestration of Na^+^ into vacuoles. Compartmentalization of Na^+^ into vacuole not only provides an efficient mechanism to avert deleterious effects of Na^+^ in cytoplasm, but also allows plant to use Na^+^ as an osmoticum, for maintaining an osmotic potential for driving water into cell [4], [7]. Vacuolar compartmentalization of Na^+^ is a critical process in salt adaptation, which is conserved in both halophytes and glycophytes. Na^+^ transport into vacuoles mediated through vacuolar Na^+^/H^+^ antiporter is an energy driven process involving H^+^ transporting pumps such as H^+^-ATPase and H^+^-PPase [8]. The genes encoding for Na^+^/H^+^ antiporters (NHX) have been cloned from more than 60 plant species, including gymnosperms and dicotyledonous and monocotyledonous angiosperms. The expression of most *NHXs* was induced by NaCl treatment [9]. Overexpression of vacuolar NHX genes suppressed the salt sensitive phenotype of a yeast mutant defective for endosomal and vacuolar Na^+^/H^+^ antiporters and conferred salt tolerance in transgenic plants [10], [11]. Several reports on improvement of salt tolerance through overexpression of vacuolar *NHX*s in agriculturally important but glycophytic crops implicate a pivotal function of the *NHX*s in intracellular compartmentalization of Na^+^ and salt tolerance [3], [12]. In legumes, *NHX1* has been reported in *Glycine max* [13], *Medicago sativa* [14], *Trifolium repens* [15], *Lotus tenuis* [16], *Caragana korshinskii* [17] and recently by our lab, in *Vigna unguiculata* (GenBank Acc. No. JN641304.2). However, no salt-tolerant genes including *NHX* yet reported from mungbean.

Mungbean (*Vigna radiata* L. Wilczek) is an important grain legume widely cultivated in south, east and south-east Asian countries for its protein rich grains. Salinity is recognized as major constraint in the production of mungbean [18], [19]. Mungbean is moderately drought tolerant [20] and therefore, this distinctive character makes it a valuable tropical crop legume for studying the molecular tolerance mechanisms for various abiotic stresses including salinity. In this paper, we report the cloning and molecular characterization of *VrNHX1* antiporter from *V. radiata*, its expression pattern under various abiotic stresses like salt, dehydration and cold stress, functional complementation of *VrNHX1* in *Saccharomyces cerevisiae* salt sensitive mutant (AXT3) and finally, increased salt tolerance by constitutive and inducible expression of *VrNHX1* in transgenic *Arabidopsis thaliana*, highlighting the potent role of *VrNHX1* in salt tolerance mechanisms.

## Materials and Methods

### Plant Material and Stress Treatment

Mungbean (*Vigna radiata* L. Wilczek cv. K-851) seeds were surface sterilized with 0.2% mercuric chloride and rinsed three times with distilled water. The seeds were germinated in dark chamber for 2 days, transferred to Hoagland's nutrient medium, grown hydroponically in a controlled growth chamber at 25°C, 80% relative humidity with a 16 hr/8 hr photoperiod and photosynthetic flux intensity of 300 µmol m^−2^ s^−1^ for 14 days. For salt stress treatment, these two weeks old mungbean seedlings grown under hydroponic culture were transferred to 200 mM NaCl solution for 12 hrs and roots were harvested, frozen immediately, and stored at −80°C until further use.

### Molecular cloning of *VrNHX1* cDNA by RACE approach

Total RNA was isolated from salt-treated roots of mungbean using AMBION RNAqueous Kit (Ambion, Carlsbad, CA, USA). One microgram of RNA was used for cDNA synthesis using Revert Aid First Strand cDNA Synthesis Kit (Thermo Fisher Scientific, Waltham, MA, USA). The cDNA was amplified with a pair of degenerate primers (Deg FP: 5′- TAT(A/T)ATATTCAATGC(C/A)GGGTTTCA(G/A)GT(A/G) -3′ and Deg RP: 5′- GCATT(A/G)TGCCA(A/G)GT(A/G)TAATG(A/T)GACAT(A/G/C)AC -3′) designed from the conserved region of transmembrane domains of plant NHX antiporters submitted in NCBI database. The PCR condition was: 94°C for 3 min; 94°C for 30 sec, 52°C for 30 sec, 72°C for 30 sec with 30 cycles, and a final extension at 72°C for 10 min. Based on the resulting partial fragment, gene specific primers were designed for amplification of 5′- and 3′- untranslated regions of *VrNHX1*.

The 5′ RACE was performed using the 5′ RACE System for Rapid Amplification of cDNA Ends Kit, Version 2.0 (Invitrogen, Carlsbad, CA, USA). Briefly, five micrograms of RNA was used for first strand cDNA synthesis using a gene specific primer (GSP1: 5′- CTGCTTCTTTTTCACCTGAAACCCAGC -3′) and Superscript II reverse transcriptase (Invitrogen). cDNA was purified using SNAP column to remove unincorporated dNTPs and primer, that might interfere in the homopolymeric tailing of cDNA. Terminal transferase enzyme was used to add dCTPs to 3′ end of cDNA. The dc-tailed cDNA was amplified using abridged anchor primer (AAP: 5′- GGCCACGCGTCGACTAGTACGGGIIGGGIIGGGIIG -3′) and gene specific primer (GSP2: 5′- ACCTGAAACCCAGCATTGAATAT-3′). The PCR condition was: 94°C for 3 min; 94°C for 30 sec, 55°C for 30 sec, 72°C for 30 sec with 30 cycles, and a final extension of 72°C for 10 min. Further, nested PCR was performed using abridged universal anchor primer (AUAP: 5′- GGCCACGCGTCGACTAGTAC -3′) and nested gene specific primer (GSP3: 5′- GGTATATGAAGAAAAGATCTTC -3′) using the first PCR product as template. The PCR condition was: 94°C for 3 min; 94°C for 30 sec, 52°C for 30 sec, 72°C for 30 sec with 35 cycles, and a final extension of 72°C for 10 min.

The 3′ RACE was performed using 3′ RACE System for Rapid Amplification of cDNA Ends Kit, Version E (Invitrogen, Carlsbad, CA, USA). Five micrograms of RNA was used to synthesize cDNA using a dT-adapter primer (AP: 5′- GGCCACGCGTCGACTAGTAC(T)_17_ -3′) and Superscript II reverse transcriptase (Invitrogen, Carlsbad, CA, USA). The first 3′-RACE-PCR was carried out using gene specific primer (GSP4: 5′- AGTGGCATCCTCACTGTATTCTTTTGTG -3′) and abridged universal anchor primer (AUAP: 5′- GGCCACGCGTCGACTAGTAC -3′). The PCR condition was: 94°C for 3 min; 94°C for 30 sec, 60°C for 30 sec, 72°C for 1 min and 30 sec with 30 cycles, and a final extension of 72°C for 10 min. The PCR product was diluted 10 times (1∶10) and used as template for nested 3′ RACE-PCR. The nested 3′-RACE-PCR was carried out using gene specific primer (GSP5: 5′-GCTGTATATTGGAAGGCACTCT-3′) and abridged universal anchor primer (AUAP: 5′-GGCCACGCGTCGACTAGTAC -3′). The PCR condition was: 94°C for 3 min; 94°C for 30 sec, 55°C for 30 sec, 72°C for 1 min and 30 sec with 30 cycles, and a final extension of 72°C for 10 min. The above PCR products were cloned to TA cloning vector pTZR/T (Thermo Fisher Scientific, Waltham, MA, USA) sequenced and contiguous sequences aligned to obtain full length of *VrNHX1* cDNA.

### Bioinformatic analysis of *VrNHX1*

Multiple sequence alignment and phylogenetic analysis were performed using Clustal W [21]. A phylogenetic tree was constructed using neighbor joining method and reliability of the tree was analyzed with bootstrap analysis with 500 replicates using MEGA4 (Molecular Evolutionary Genetics Analysis): Tree Explorer software [22]. Hydrophobicity plot and transmembrane domain prediction was performed using TMpred software [23]. Post-translational modification of *VrNHX1* was predicted by searching for conserved motifs of N- and O- glycosylation and N-myristoylation sites using ScanProsite [24].

### Southern hybridization for *VrNHX1* copy number in mungbean genome

Twenty µg of genomic DNA was used for gene copy analysis of *VrNHX1* and digested with restriction endonucleases EcoRI and HindIII. Digested genomic DNA was electrophoretically fractionated on 0.8% agarose gel and blotted onto Zeta-Probe membrane (Bio-Rad, Hercules, CA, USA). The blot was hybridized with DIG-labeled 1.6 kb PCR product, corresponding to the coding region of *VrNHX1*. Southern hybridization was carried out using solution containing 50% formamide, 5 X SSC, 5 X Denhardt's solution, 0.05 M sodium phosphate pH 6.5, 0.1% SDS, 10% dextran sulfate, 0.1 mg/ml sheared denatured salmon-sperm DNA and 20 ng/ml probe at 42°C for 18 hrs. Washing and detection was performed according to instructions of the DIG Labeling and Detection system (Roche Diagnostics, Mannheim, Germany).

### Heterologous expression of *VrNHX1* in yeast mutant

Functional complementation assay was performed in yeast strains, W303-1B (*MATα ade2-1 can1-100 his3-11,15 leu2-3,112 trp1-1 ura3-1*) and AXT3 *(Δ ena1- 4::HIS3 Δnha1::LEU2 Δnhx1::TRP1*, *ura3-1)*. Yeast strains were grown in YPD (1% Yeast extract, 2% Peptone and 2% Glucose), YPGal (1% Yeast extract, 2% Peptone and 2% Galactose), SC (0.67% Yeast Nitrogen Base, 2% Glucose) and APGal synthetic minimal media (10 mM arginine, 8 mM phosphoric acid, 2 mM MgSO_4_, 1 mM KCl, 0.2 mM CaCl_2_, 2% Galactose, trace vitamins, and minerals; pH-4.0) supplemented with appropriate amino acids as indicated.

The CDS of *VrNHX1* was cloned into yeast expression vector pYES2.0 (Invitrogen, Carlsbad, CA, USA) with restriction sites of KpnI and BamHI. The yeast strains were transformed with pYES2.0 empty vector (labeled as AXTYES2.0 strain) or pYES*VrNHX1* recombinant plasmid (labeled as AXTVrNHX1 strain) by Lithium acetate method [25] and selected on SC ura^−^ medium.

For growth assay, precultured cells were grown till OD_600_ of 1.0, diluted to an OD_600_ of 0.006, and inoculated to liquid APGal ura^−^ synthetic minimal media supplemented with different concentrations of NaCl, KCl, and LiCl and grown at 30°C for 48 hrs. For complementation assay, saturated liquid cultures (OD_600_ 0.8) of each strain were serially diluted to 10, 100 and 1000 fold and spotted on APGal solid media supplemented with or without 50, 75 and 100 mM NaCl, 0.5 M KCl, 25 mM LiCl and and YPGal media supplemented with 50 µg/ml hygromycin. Plates were maintained at 30°C. Growth was monitored after 3 days.

### Intracellular measurement of Na^+^ and K^+^ distribution in yeast mutant

Intracellular ion was extracted from yeast strains grown in liquid APGal media, pH 4.0 supplemented without or with 75 mM NaCl [26]. Briefly, cells were harvested at an OD_600_ of 0.3–0.4, centrifuged at 3000 g/3 min, washed twice in ice-cold 10 mM MgCl_2_, 10 mM CaCl_2_ and 1 mM HEPES buffer and resuspended in the same buffer. The relationship between cell density (Absorbance at OD_600_) and yeast dry weight was determined. Total intracellular ion was determined by addition of HCl to a final concentration of 0.4% and incubated at 95°C for 20 min. After removal of cell debris the supernatant was measured for presence of Na^+^ and K^+^. Similarly, cells were grown and washed as above and resuspended in 2% cytochrome c, 18 µg/ml antimycin, 1 mM HEPES, 10 mM MgSO_4_, 10 mM CaCl_2_, and 5 mM 2-Deoxy D-Glucose solution. Cytochrome c selectively permeabilizes the plasma membrane. After 20 min incubation at room temperature, cells were washed thrice with the same solution without cytochrome c. Cytoplasmic ion content was determined by pooling the supernatants. The remaining vacuolar ions were extracted with addition of HCl in a final concentration of 0.4% and incubated at 95°C for 20 min. The Na^+^ and K^+^ distribution in the cytoplasmic and vacuolar fractions were measured in flame photometer (Systronics, MP, India).

### Vacuolar pH estimation and fluorescence imaging

Yeast cells were grown in APGal medium (pH 5.0) to an OD_600_: 0.25–0.3, pelleted, and washed with deionized distilled water. Further, the yeast cells were incubated with 50 µM 2′,7′-bis-(2-carboxyethyl)-5-(and-6)-carboxyfluorescein (BCECF-AM) (Molecular Probes, Eugene, Oregon) for 30 min, centrifuged, washed thrice and resuspended in APGal medium (pH 5.0) and immediately used for fluorescence measurement. Single emission fluorescence measurement at 490 nm excitation wavelength and absorbance at 600 nm were measured using LS 55 Fluorescence Spectrophotometer (Perkin Elmer, Waltham, MA, USA). The calibration curve for fluorescence intensities at different pH was obtained for each strain [27]. Briefly, the yeast strains (W303-1B, AXTYES2.0, AXTVrNHX1) were incubated in experimental medium containing 50 mM MES, 50 mM HEPES, 50 mM KCl, 50 mM NaCl, 0.2 M ammonium acetate, 10 mM NaN_3_, 10 mM 2- deoxy glucose, 50 µM carbonyl cyanide m-chlorophenylhydrazone, titrated to five different pH values within the range of 4.0 to 8.0. Background subtracted I_490_ values were normalized to cell density for each strain, labeled as NI_490_ and plotted against pH values. For vacuolar pH estimation, experimental NI_490_ values corresponding to each strain was analyzed with the calibration curve specific for each strain.

For vacuolar pH imaging the yeast cells were grown, pelleted to be suspended in the same medium with 50 µM BCECF-AM pH specific dye as above. For fluorescence imaging, 100 µl of BCECF-loaded yeast suspension was plated onto glass cover slips precoated with concavalin-A (Sigma-Aldrich, St. Louis, MO, USA) and placed on glass slides. Fluorescence images were captured in Nikon eclipse Ti-U Fluorescence microscope (Nikon, Chiyoda, Tokyo, Japan).

### Expression analysis of *VrNHX1* using semi-quantitative RT-PCR

Expression analysis under salt stress: Two different stages of growth in mungbean seedlings i.e. early and mid stage, were considered for expression analysis under salt stress (200 mM NaCl). Mungbean seedlings were germinated, grown in Hoagland's nutrient medium for five and ten days, in case of early and mid stage respectively, and transferred to 200 mM NaCl solution for salt stress assay. Leaves and roots of salt treated early and mid stage mungbean seedlings, were harvested at time intervals 0, 6, 12, 18, 24, and 48 hrs. Similarly, expression pattern for *VrNHX1* in response to different forms of abiotic stress such as salt (200 mM NaCl), dehydration (200 mM mannitol) and cold stress (4°C) was also studied at different time intervals (0, 6, 12, and 24 hrs) for mid-stage (10 days old) mungbean seedlings. Total RNA was extracted using RNeasy Plant Mini Kit (Qiagen, Venlo, Limburg, Netherlands) and reverse transcribed using Revert Aid First Strand cDNA Synthesis Kit. Semi-quantitative RT-PCR was performed using gene specific primers (RF: 5′- GTATTTCCACTGGCGTAGTCATTTTGC -3′ and RR: 5′- GCATCATTCACAGCACCCTCTCGG -3′). The PCR condition was: 94°C for 3 min; 94°C for 30 sec, 62°C for 30 sec, 72°C for 30 sec for 28 cycles, and a final extension at 72°C for 10 min. Housekeeping *VrTubulin-β* primers (FN: 5′- CTTGACTGCATCTGCTATGTTCAG-3′ and RN: 5′-CCAGCTAATGCTCGGCATACTG -3′) were used as an internal control. The PCR condition was: 94°C for 3 min; 94°C for 30 sec, 58°C for 30 sec, 72°C for 30 sec for 28 cycles, and a final extension at 72°C for 10 min. Semi-quantitative RT-PCR was repeated three times. The PCR products were analyzed in 2% agarose gel stained with 10 mg/ml ethidium bromide.

### Measurement of total ion content in salt stressed mungbean seedlings

Leaves and roots of untreated and salt-treated early and mid stage mungbean seedlings were harvested at different time intervals (0, 6, 12, 18, 24, 48 and 72 hrs). The samples were dried, digested with concentrated HNO_3_ at 90°C for 1 hr and centrifuged at 12,000 rpm for 10 min [28]. The suspension was diluted with sterile milliQ water and analyzed for Na^+^ and K^+^ content in flame photometer.

### Binary vector preparation and plant transformation

The 1.6 kb CDS of *VrNHX1* was cloned into standard plant binary vector pCAMBIA2301 (11.6 kb) flanked by cauliflower mosaic virus CaMV 35S promoter and terminator at *Pst*I restriction site. The resulting recombinant plant binary vector was labeled as pCAMBIA2301-35S::*VrNHX1* (13.9 kb). Further, a 0.898 kb promoter fragment of *AtRD29A* (DQ071887.1) was amplified from *A. thaliana* genomic DNA and cloned into *Eco*RI digested recombinant binary vector pCAMBIA2301-35S::*VrNHX1* (13.9 kb) by replacing the 0.4 kb 35S promoter fragment from 35SP::*VrNHX1*::35STer cassette. The resulting binary vector was named pCAMBIA2301-RD29A::*VrNHX1* (14.4 kb).

The recombinant plant binary vectors, pCAMBIA2301-35S::*VrNHX1* (13.9 kb) and pCAMBIA2301-RD29A::*VrNHX1* (14.4 kb) were transferred into *A. tumefaciens* GV3101 strain via electroporation at 1250 V with capacitance of 25 mF and resistance of 400 ohm. The constructs were used for transformation of *Arabidopsis thaliana* (ecotype Columbia) via floral dipping method [29]. The T_1_ transgenic lines were screened on ½ MS medium (Duchefa, Haarlem, Netherlands) supplemented with 50 mg/l kanamycin (Duchefa, Haarlem, Netherlands). The transgenic selections were continued until T_3_ generation to obtain homozygote transgenic lines with a single T-DNA locus (35S::*VrNHX1* or RD29A::*VrNHX1*).

### RNA extraction and Real Time PCR of transgenic *Arabidopsis* lines

Total RNA was extracted from wild-type (WT) and T_3_ independent 35S::*VrNHX1* and RD29A::*VrNHX1* transgenic lines using RNeasy Plant Mini Kit (Qiagen), quantified in Nanovue Plus Spectrophotometer (GE Healthcare, Little Chalfont, Buckinghamshire, UK) and cDNA was prepared using Revert Aid First Strand cDNA Synthesis Kit. The gene specific forward primer (VrRTF: 5′- TGATTCAATCCATCGACCAA-3′) and 35S poly-A reverse primer (TerparR: 5′-GCGAAACCCTATAAGAACCCTAATTCC-3′) were used for amplification of a 0.283 kb fragment of *VrNHX1*::35S poly-A in transgenic *A. thaliana* plants. Housekeeping (UBQ1FP: 5′- AGAGCTGTCAACTGCAGGAAGAA-3′ and UBQ1RP- 5′-ACAAGAAAAACAAACCCTATCAAA GG) primers were used to amplify a 150 bp fragment of *AtUbiquitin* to be used as an internal control. Real time PCR was performed using USB VeriQuest SYBR Green qPCR Master Mix (2X) (Affymetrix, Santa Clara, CA, USA) and primers at a final concentration of 200 nM in 7500 Real-Time PCR System (Applied Biosystem, Foster City, California, USA) following the manufacturer's protocol. The experiment was repeated twice independently with three replicates. The expression values relative to the standard curve was calculated for each sample. The relative expression level of transgene *VrNHX1* in wild-type (WT) and transgenic 35S::*VrNHX1* and RD29A::*VrNHX1 Arabidopsis* lines was estimated by normalizing *VrNHX1* expression values with respect to housekeeping *AtUBQ1* expression values in each case.

### Salt tolerance assays of transgenic *Arabidopsis* lines

Wild-type (WT) and T_3_ transgenic 35S::*VrNHX1* and RD29A::*VrNHX1 Arabidopsis* seeds were germinated on ½ MS medium [30] in growth chamber maintained at 22°C and 60% relative humidity with a 16 hr/8 hr photoperiod under controlled conditions.

Studying germination efficiency under salt stress: The WT and T_3_ transgenic 35S::*VrNHX1* and RD29A::*VrNHX1* lines were germinated on ½ MS medium supplemented with or without 150 mM NaCl and kept at 4°C for 3 days, prior to, transfer to growth chamber. The germination efficiency was studied after 10 days of salt stress.

Measurement of growth parameters under salt stress: The 4 days old germinated seedlings were transferred to ½ MS medium supplemented with or without 150 mM NaCl for 1 week and the difference in root length of wild-type WT and T_3_ independent transgenic lines of *Arabidopsis* seedlings expressing *VrNHX1* was measured. Mean data was collected from ten replicates (n = 10) for wild-type (WT) and T_3_ kanamycin selected transgenic *Arabidopsis* lines.

Measurement of physiological parameters under salt stress: The 10 days old germinated seedlings were transferred to ½ MS liquid medium supplemented with or without 200 mM NaCl for 5 days. For measurement of chlorophyll content, shoot samples were homogenized in 95% ethanol, lysate was centrifuged at 3,000 rpm for 10 min and absorbance was recorded for the extract at wavelength of 648 and 664 nm [31]. Lipid peroxidation was measured as the amount of malondialdehyde (MDA) determined by the thiobarbituric acid (TBA) reaction. Briefly, 0.2 g of fresh leaf samples were homogenized with 5 ml of 0.25% TBA containing 10% TCA (tricloroacetic acid). The homogenate was boiled for 30 min at 95°C and centrifuged at 10,000 g for 10 min Absorbance values were recorded at 532 nm and values corresponding to non-specific absorption at 600 nm were subtracted [32]. For colorimetric estimation of proline, leaf samples (0.5 g) were homogenized with 5.0 ml of sulfosalicylic acid (3%). 2 ml of homogenate was filtered through Whatman filter paper (No. 2) and incubated with 2 ml glacial acetic acid and 2 ml ninhydrin reagent at a ratio of 1∶1∶1 in boiling water bath at 100°C for 30 min. After cooling, 4 ml toluene was added to the reaction mixture, mixed vigorously and absorbance was measured at 520 nm [33]. Mean data was collected from three replicates (n = 3) for wild-type and T_3_ kanamycin selected transgenic *Arabidopsis* lines.

### Measurement of Na^+^ and K^+^ in transgenic *Arabidopsis* lines

The germinated seedlings were initially grown in ½ MS medium (0.5% agar) for 5 days and then subsequently transferred to soilrite and grown for 2 weeks. The WT and T_3_ transgenic lines were subjected to salt stress for a period of 2 weeks by watering them with ½ MS nutrient liquid media supplemented with 250 mM NaCl. The whole plant was harvested for Na^+^ and K^+^ estimation using method described elsewhere [30]. Mean data was collected from three replicates (n = 3) for wild-type (WT) and T_3_ kanamycin selected transgenic *Arabidopsis* lines.

### Statistical analysis

Statistical comparison between the variances was determined by ANOVA (Analysis of variance) and significant differences between mean values were determined by Bonferroni analysis. Statistically significant mean values were denoted as “*” (P≤0.05).

## Results

### Isolation and in-silico analysis of *VrNHX1*

**Figure 1:**
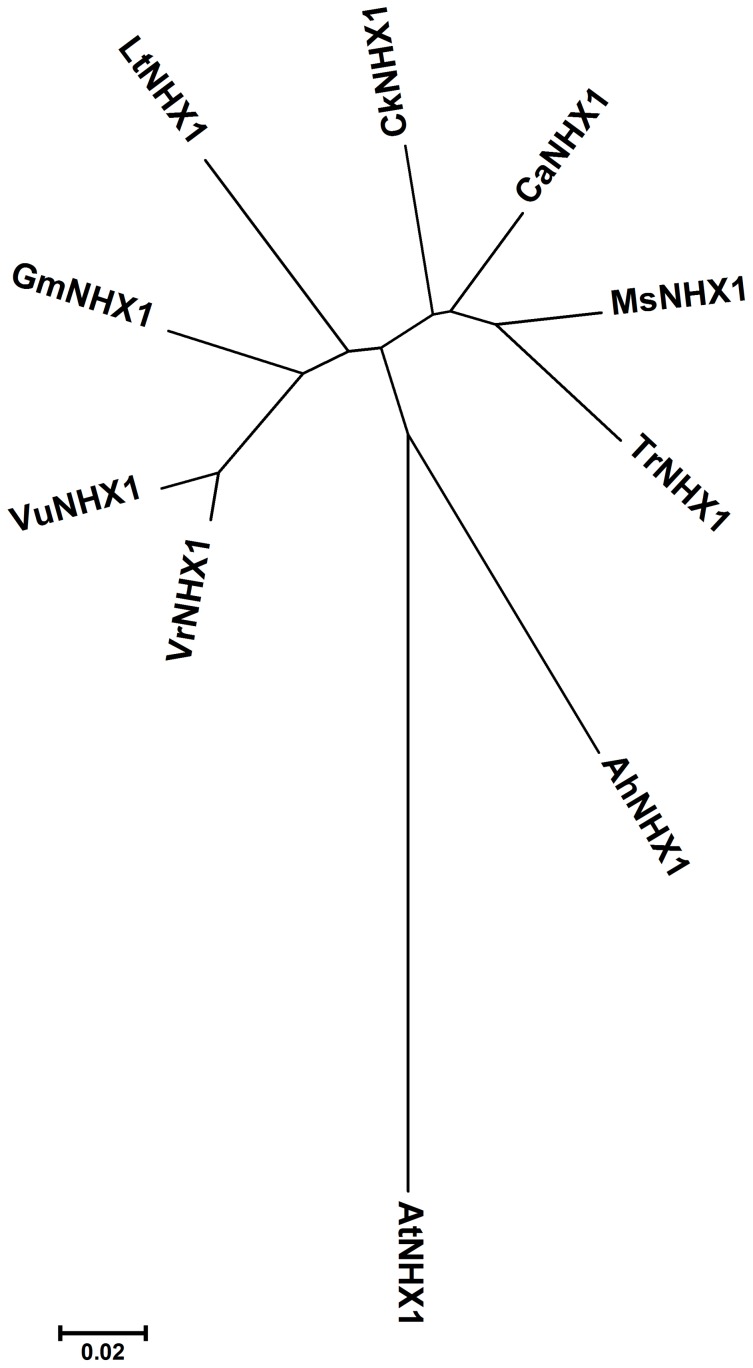
The phylogenetic tree for plant Na^+^/H^+^ antiporters was generated using MEGA4: Tree Explorer software. The evolutionary history was inferred using the neighbor-joining method and analyzed using bootstrap analysis with 500 replicates. Branches corresponding to partitions reproduced in less than 50% bootstrap replicates are collapsed. The tree is drawn to scale, with branch lengths in the same units as those of the evolutionary distances used to infer the phylogenetic tree. The evolutionary distances were computed using the Poisson correction method and are in the units of the number of amino acid substitutions per site. The GenBank Accession numbers for NHX proteins used are: VrNHX1 (AEO50758.1), VuNHX1 (AEO72079.2), GmNHX1 (AAY430061.1), CkNHX1 (ABG89337.1), MsNHX1 (AAS84487.1), CaNHX1 (ADL28385.1), TrNHX1 (ABV00895.1), LtNHX1 (ACE78322.1), AhNHX1 (ADK74832.1), AtNHX1 (NM_122597.2).

A *VrNHX1* cDNA of 2095 nucleotides in length (Genbank Accession number JN656211.1), with an open reading frame of 1,629 bp was obtained by RACE-PCR approach. It encodes a polypeptide of 542 amino acid residues with an estimated molecular mass 59.60 kDa and isoelectric point 6.76, predicted using ExPaSy bioinformatic tools for protein structure analysis (http://www.expasy.org/tools/). Multiple sequence alignment of deduced amino acid sequences of VrNHX1 revealed that it has 97.42% sequence identity with *Vigna unguiculata*, 92.25% with *Glycine max*, 88.48% with *Caragana korshinskii*, 87.27% with *Lotus tenuis*, 87.25% with *Trifolium repens*, 87.06% with *Medicago sativa*, and 86.72% with *Cicer arietinum* (Fig. 1 and S1). Phylogenetic relationship analysis performed using MEGA4 software indicated that VrNHX1 clustered into Class-I type IC-NHX legume *NHX* homologs, more closely to VuNHX1 and GmMHX1 (Fig. 1). The hydropathy plot of VrNHX1 protein predicted by TMpred software indicated highly hydrophobic N-terminal end with 11 putative transmembrane domains and a longer hydrophilic C-terminal end inside the vacuolar lumen (Fig. S2). The amiloride binding motif, ^84^-LFFIYLLPPI-^93^, a classic inhibitor of Na^+^/H^+^ antiporters [34] and also highly conserved among eukaryotic Na^+^/H^+^ exchangers, was detected in TM3 region (Fig. S1). The prediction of putative post-translational modification sites by ScanProsite software indicated presence of two potential *N-*glycosylation (ASN_glycosylation) sites, fifteen phosphorylation sites for protein kinase CK2 and protein kinase C, ten *N-*myristoylation sites, and one Leucine Zipper site (Table S1).

**Figure 2:**
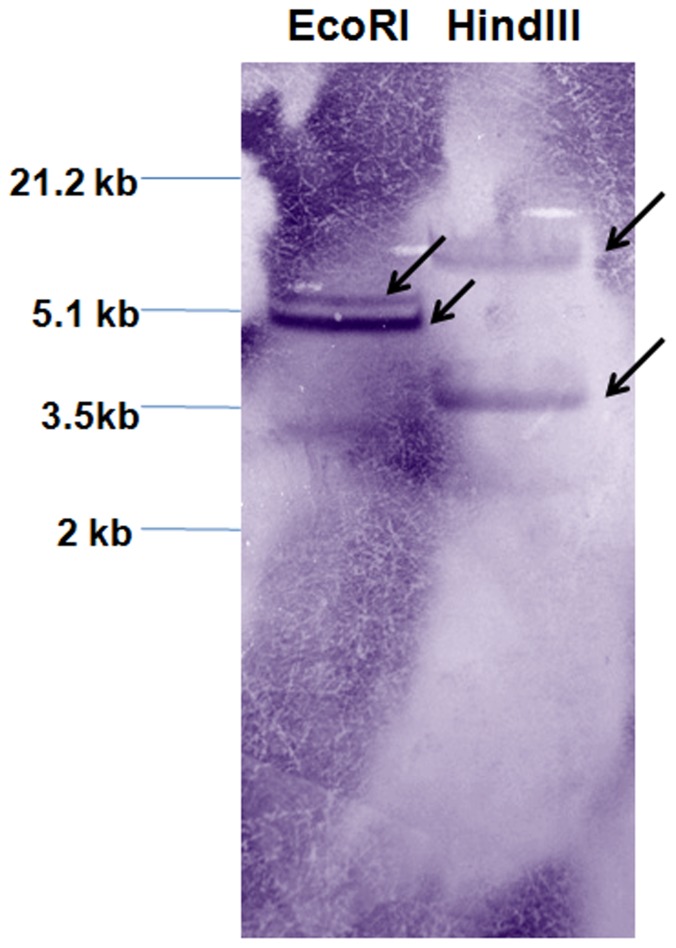
Copy number analysis of *VrNHX1* in mungbean genome. Mungbean genomic DNA (20 µg) was digested with EcoRI and HindIII, and hybridized with DIG-labeled probe corresponding to the CDS of *VrNHX1*. Hybridization signals are indicated as arrows.

The Southern hybridization analysis revealed presence of single copy of *VrNHX1* in mungbean genome (Fig. 2). Two hybridization signals, one each for HindIII and EcoRI digested mungbean genome were detected, possibly due to the occurrence of a single *Hind*III site in *VrNHX1* (1.6 kb). Occurrence of a single *Eco*RI site in genome fragment of *VrNHX1* was accounted for getting two signals as probe lacked any *Eco*RI site.

### Functional characterization of *VrNHX1* using salt sensitive yeast mutant

**Figure 3:**
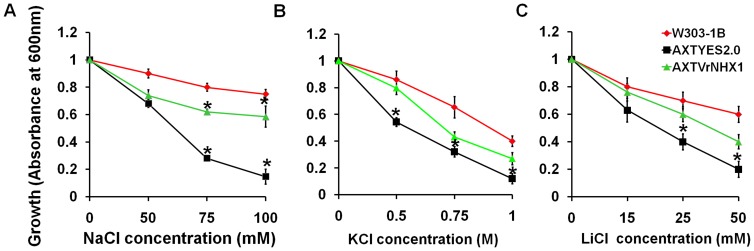
Cation sensitivity assay of transformed yeast strains (W303-1B, AXTYES2.0, AXTVrNHX1) under various concentrations of NaCl, KCl, and LiCl. Saturated seed cultures for each strain was diluted to an OD_600_ of 0.006 and inoculated to liquid APGal medium (pH 5.5) supplemented with or without various concentrations of (A) NaCl (0, 50, 75, 100 mM), B) KCl (0, 0.5, 0.75, 1.0 M), and (C) LiCl (0, 15, 20, 25 mM). Growth was observed at 30°C after 3 days and absorbance recorded at 600 nm. Data are means of 3 independent events (n = 3) and standard errors are plotted in the graph. Statistically significant values at P≤0.05 are indicated as “*”, using Bonferroni analysis.

**Figure 4:**
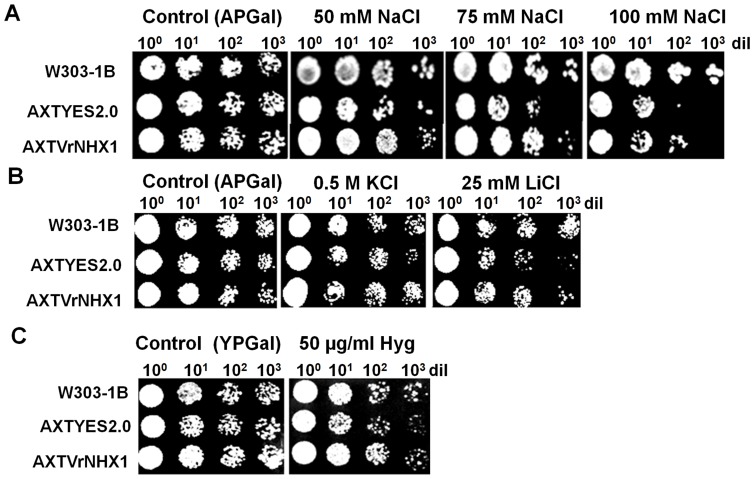
Heterologous expression of *VrNHX1* in yeast mutant. Wild type (W303-1B) strain was used as a control, *Δ ena1- 4 Δnha1 Δnhx1* mutant (AXT3) strain was transformed with null pYES2.0 (labeled as AXTYES2.0 strain) and pYES*VrNHX1* recombinant vector (labeled as AXTVrNHX1) were used for complementation assay. 10-fold serial dilutions of saturated seed cultures of each strain were spotted onto APGal media (pH-5.5) supplemented with or without (A) 50, 75 and 100 mM NaCl, (B) 25 mM LiCl, and (B) 0.5 M KCl. (C) Hygromycin sensitivity assay was performed by spotting 10-fold serial dilutions of saturated seed cultures of each strain onto YPGal media (pH- 5.5) supplemented with or without 50 µg/ml Hyg. The plates were incubated at 30°C for 3 days.

Previous work showed that heterologous expression of Na^+^/H^+^ antiporter genes in yeast mutant AXT3 could partly suppress its hypersensitivity to hygromycin and restore salt tolerance. The similar method was exploited to initially characterize the function of *VrNHX1*. The AXTVrNHX1 cells displayed enhanced Na^+^, K^+^ and Li^+^ tolerance with statistically significant improvement in their survival at NaCl (75 and 100 mM) (Fig. 3 A) and 0.5 M KCl (Fig. 3 B), in contrast to AXTYES2.0 cells. Expression of *VrNHX1* in AXT3 cells under GAL1-inducible promoter restored salt tolerance upto 100 times dilution in 75 and 100 mM NaCl (Fig. 4 A), and better survival at 1000 times dilution range in 25 mM LiCl and 0.5 M KCl in AXTVrNHX1 cells on solid media (Fig. 4 B). *ScNHX1* has been suggested to ameliorate sensitivity of yeast cells by sequestering hygromycin-B, a cationic aminoglycoside antibiotic in vacuole. Therefore, yeast mutant lacking *NHX1* is more susceptible to hygromycin treatment [27]. *VrNHX1* expression showed suppression of hygromycin (50 µg/ml) sensitivity in AXTVrNHX1 cells (Fig. 4 C).

### Na^+^ and K^+^ distribution in yeast mutants

**Figure 5:**
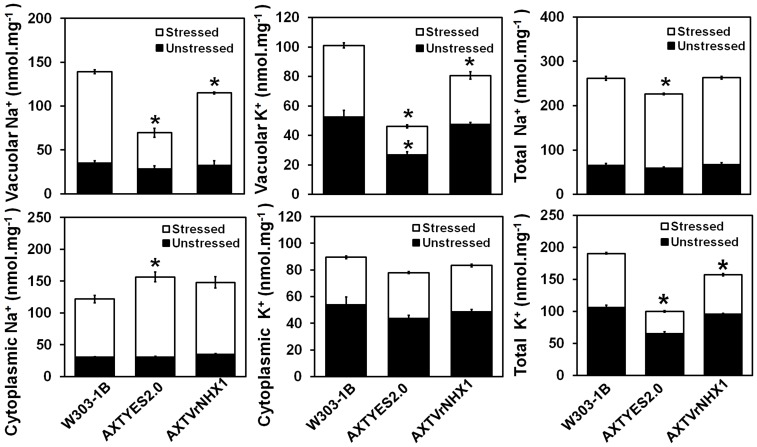
Total intracellular ion estimation in yeast strains W303-1B, AXTYES2.0 and AXTVrNHX1. Yeast cells were grown in APG medium (pH 4.0) with 1 mM KCl supplemented in presence (stressed) or absence of 75 mM NaCl (unstressed) and harvested at a cell density of 0.3. Total intracellular, vacuolar and cytoplasmic Na^+^ and K^+^ content was determined as described in the materials and methods section. Data are means of 3 independent events (n = 3) and standard errors are plotted in the graph. Statistically significant values at P≤0.05 are indicated as “*”, using Bonferroni analysis.

The AXTYES2.0 cells displayed 2.3 times lower K^+^ content than AXTVrNHX1 cells under normal condition owing to lack of yeast Na^+^/K^+^/H^+^ antiporter activity (Fig. 5). Under salt stress, AXTVrNHX1 cells accumulated 2 times higher and 4.8 times lower vacuolar Na^+^ content compared to AXTYES2.0 and W303-1B cells, respectively (Fig. 5). Similarly, vacuolar K^+^ content observed for AXTVrNHX1 cells was 2.36 times higher than AXTYES2.0 cells. The cytoplsamic Na^+^ content was higher in both the cell types as compared to W303-1B, due to the loss of NHA exchanger activity which cannot be solely compensated by *VrNHX1* complementation. However, cytoplasmic K^+^ fractions measured were not statistically significant, though AXTVrNHX1 cells exhibited higher K^+^ values as compared to AXTYES2.0, indicating the improved ability of AXTVrNHX1 cells in maintaining a higher intracellular K^+^/Na^+^ ratio for ionic homeostasis. The total ion content in yeast cells was in accordance with distribution of Na^+^ and K^+^ in cytoplasm and vacuole.

### Vacuolar pH estimation and imaging

**Figure 6:**
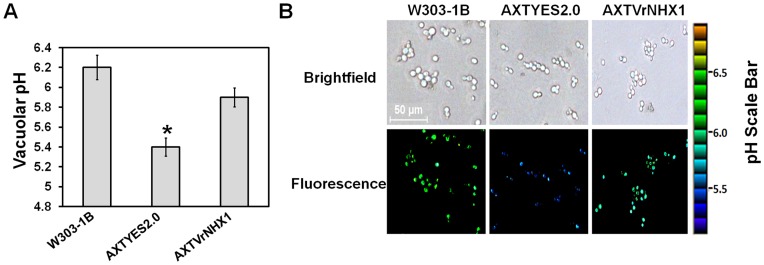
Measurement of vacuolar pH in yeast strains. (A) Vacuolar pH was measured for BCECF-AM loaded yeast strains W303-1B, AXTYES2.0 and AXTVrNHX1 as described in materials and methods following the calibration curve (Figure S4). Mean and SEs are plotted for three independent events (n = 3) in each case. Statistically significant values at P≤0.05 are indicated as “*”, using Bonferroni analysis. (B) Accumulation of pH-sensitive fluorescent BCECF dye in yeast vacuoles was measured. The yeast strains were grown in APGal media (pH 5.0), resuspended in minimal medium with BCECF-AM dye for 30 min at 30°C. Yeast cells were visualized by Nikon eclipse Ti-U Fluorescence microscope (Nikon) at excitation wavelength of 440 nm. Bar scale: 50 µm.

2′,7′-bis-(2-carboxyethyl)-5-(and-6)-carboxyfluorescein (BCECF-AM), a widely used cell-permeant and pH-sensitive fluorescent indicator was used to measure the change in vacuolar pH of yeast mutant expressing *VrNHX1* grown under low pH environment. The study on the effect of low pH on growth efficiency of yeast cells showed that growth of AXTYES2.0 cells was highly affected with a 70.66% reduction in growth as compared to W303-1B. Moreover, AXTVrNHX1 mutant showed improved growth under acidic condition (Fig. S3). Vacuolar pH was estimated following calibration curve plotted for each strain (Fig. S4). An acidic vacuolar pH of 5.4 was observed for AXTYES2.0 cells whereas, a pH value 5.9 and 6.2 was recorded for AXTVrNHX1 and W303-1B cells, respectively in response to low pH stress condition (Fig. 6 A). Similarly, fluorescence images provided acidic vacuolar pH values for AXTYES2.0 cells and expression of *VrNHX1* alkalinized the vacuolar compartment (Fig. 6 B).

### Expression pattern of *VrNHX1* under abiotic stress by Semi-quantitative RT-PCR

**Figure 7:**
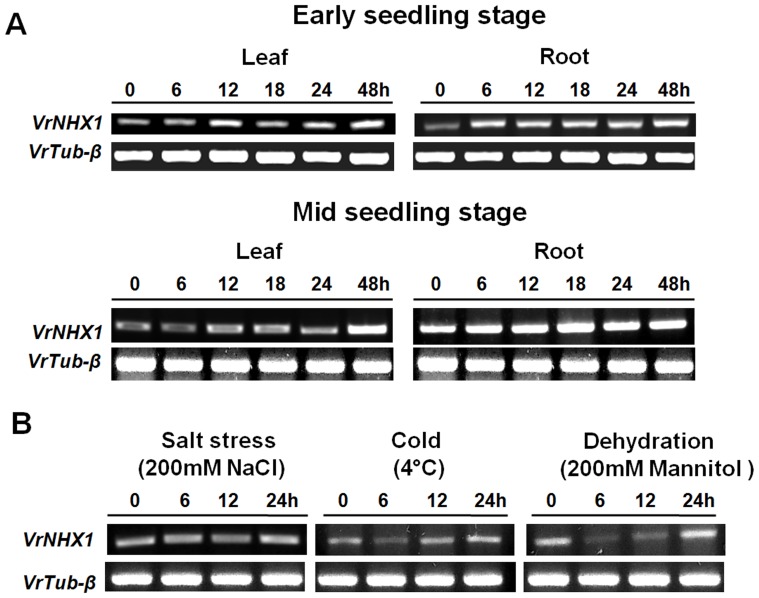
Expression analysis of *VrNHX1* in early and mid stage mungbean seedlings under various abiotic stresses. (A) Semi-quantitative RT-PCR for studying expression patterns of *VrNHX1* under salt stress was performed. Total RNA was isolated from leaves and roots of early (5 days) and mid stage mungbean seedlings (10 days) under 200 mM NaCl treatment at time intervals of 0, 6, 12, 18, 24, and 48 hrs. (B) Semi-quantitative RT-PCR for studying expression patterns of *VrNHX1* under different abiotic stress conditions such as salt, cold and dehydration stress was studied. Total RNA was isolated from mid stage mungbean seedlings under (A) 200 mM NaCl, (B) Cold (4°C), and (C) 200 mM Mannitol treatment at time intervals of 0, 6, 12, and 24 hrs. PCR fragments of 566 bp and 422 bp size corresponding to *VrNHX1* and *VrTubβ* were fractionated electrophoretically on 2% agarose gel stained with 10 mg/ml ethidium bromide.

The expression of *VrNHX1* was studied by semi-quantitative RT-PCR, in roots and leaves of mungbean seedlings at early (five days old) and mid (ten days old) growth stages exposed to salt stress (200 mM NaCl) for different time interval (0, 6, 12, 18, 24 and 48 hrs). The results indicated that transcript levels of *VrNHX1* were induced by NaCl in both roots and shoots of early and mid stage mungbean seedlings, indicating the potent role of *VrNHX1* in salt tolerance mechanisms in mungbean. In case of early seedling stage, higher expression level of *VrNHX1* was observed in leaves at 12, 24, and 48 hrs and in roots after 6 hrs (Fig. 7 A). The differential expression of *VrNHX1* in roots and leaves was also observed in mid stage seedlings, with a significant accumulation observed at 48 hrs in leaves whereas, some basal level of *VrNHX1* transcript was observed in roots under normal condition which further increased steadily with salt stress treatment period (Fig. 7 A).

To determine whether the expression of *VrNHX1* was also induced by dehydration (200 mM Mannitol) and cold (4°C), mid-stage (10 days old) seedlings were given the respective stress treatments for different time intervals (0, 6, 12, and 24 hrs). The *VrNHX1* expression varied with salt, cold and drought stress. The accumulation of *VrNHX1* transcript under salt, cold and dehydration stress reached its peak at 24 hours (Fig. 7 B). The results indicated that osmotic and low temperature stress is involved in the up-regulation of *VrNHX1* in addition to an ion-specific signaling component in mungbean. The *VrNHX1* expression analysis revealed involvement of cross talk between salinity, low temperature and osmotic stress in mungbean.

### Na^+^ and K^+^ measurement in salt stressed mungbean seedlings

**Figure 8:**
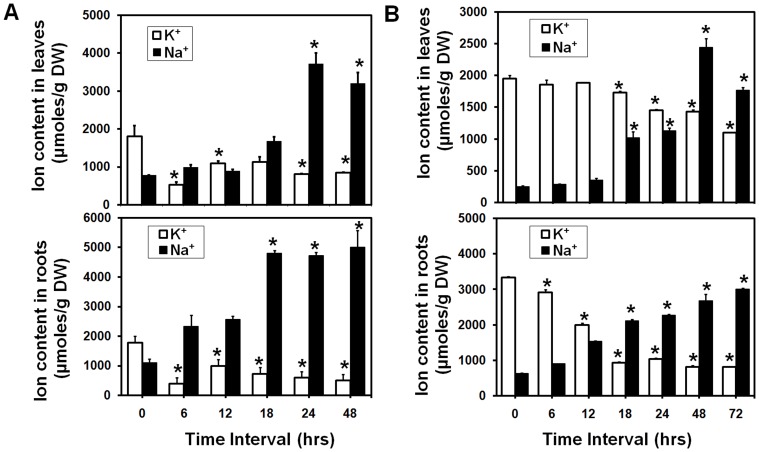
Total intracellular ion measurement in leaves and roots of early and mid stage mungbean seedlings. Na^+^ and K^+^ content in (A) leaves and (B) roots of unstressed and salt stressed mungbean seedlings harvested at time intervals of 0, 6, 12, 24, 48, and 72 hrs was measured using Flame Photometer. Values indicate means ± SE (n = 3). Statistically significant values at P≤0.05 are indicated as “*”, using Bonferroni analysis.

The measurement of Na^+^ and K^+^ content in leaves and roots of untreated and salt-treated mungbean seedlings at different time intervals (0, 6, 12 18, 24, 48 and 72 hrs) showed that under salt stress, Na^+^ accumulation increased in leaves/roots by 1.28/2.1, 1.1/2.3, 2.1/4.36, 4.8/4.3, 4.1/4.54 times whereas, K^+^ accumulation decreased by 3.4/4.5, 1.6/1.78, 1.59/2.43, 2.2/3 and 2.1/3.5 times as compared to control condition at 6, 12, 18, 24 and 48 hrs, respectively in early stage mungbean seedlings (Fig. 8 A). Similarly, in mid stage seedlings, Na^+^ accumulation in leaves/roots also increased by 1.1/1.4, 1.4/2.4, 4/3.3, 4.5/3.5, 9.8/4.2 and 7.1/4.7 times whereas, K^+^ accumulation decreased by 1.05/1.1, 1.03/1.66, 1.1/3.57, 1.34/3.2, 1.36/4.07, 1.77/4.03 times as compared to control condition at 6, 12, 18, 24, 48 and 72 hrs, respectively (Fig. 8 B). The overall higher accumulation of Na^+^ (µmoles/g DW) in roots as opposed to leaves indicated the restriction of movement of toxic Na^+^ to the aerial part of the plant as a plausible mechanism to confer salinity tolerance in mungbean.

### Ectopic expression of *VrNHX1* resulted in enhanced salt tolerance in transgenic *Arabidopsis*

**Figure 9:**
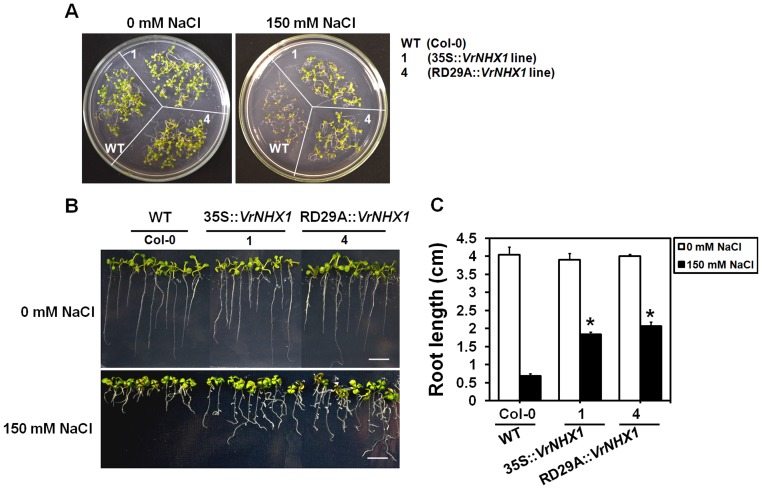
Effect of salt stress on germination efficiency and root growth of transgenic *Arabidopsis* lines. (A) The wildtype (WT, col-0) and transgenic (line 1, 35S::*VrNHX1* and line 4, RD29A::*VrNHX1*) seedlings were observed for germination score after 10 days exposure to salt stress (150 mM NaCl). (B) Root growth inhibition in wild type (WT, Col-0) and transgenic *Arabidopsis* (Line 1, 35S::*VrNHX1* and Line 4, RD29A::*VrNHX1*) plants upon salt stress (150 mM NaCl) was studied. The 4 days old germinated seedlings were transferred to 150 mM NaCl stress for a period of 7 days and (C) root length measured was plotted in graph. Values indicate means ± SE (n = 10). Statistically significant values at P≤0.05 are indicated as “*”, using Bonferroni analysis.

**Figure 10:**
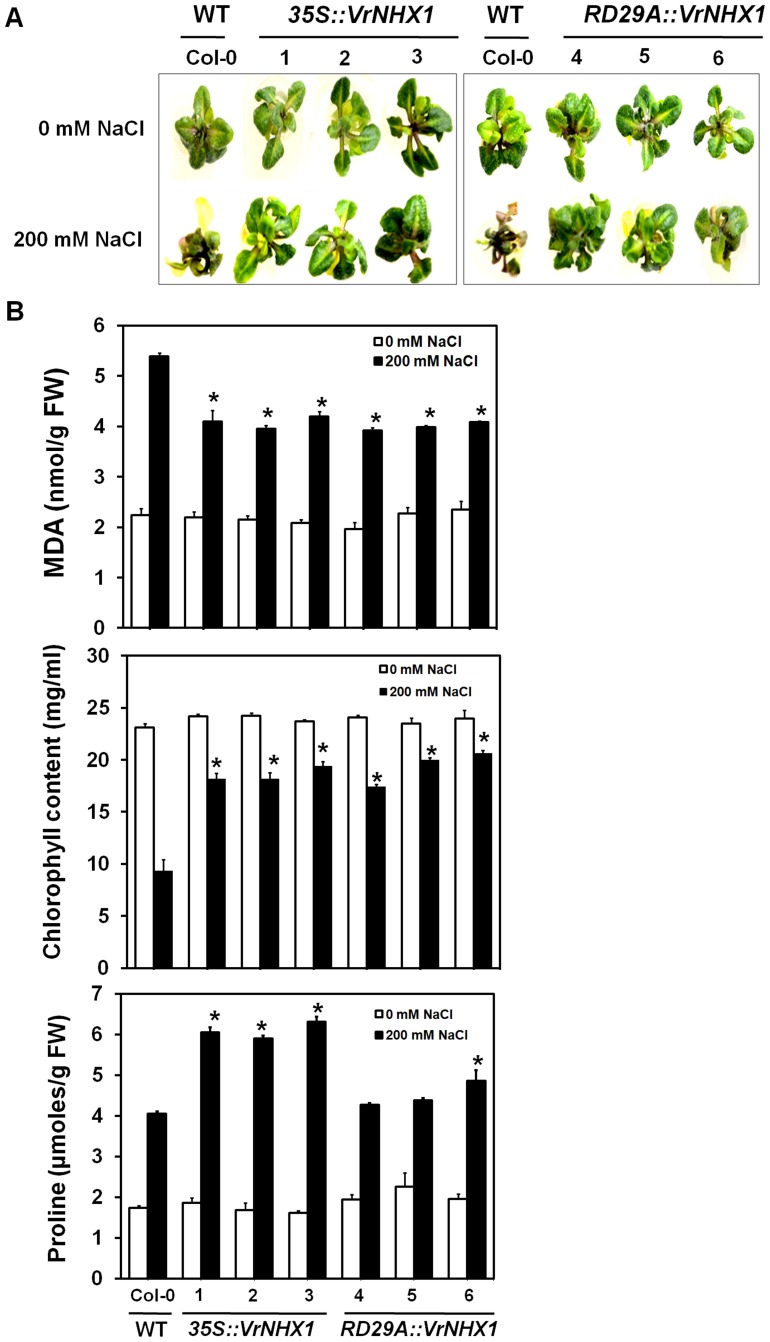
Studying the physiological changes in transgenic *Arabidopsis* lines under salt stress. (A) Effect of salt stress in wild type (WT, Col-0) and transgenic *Arabidopsis* lines expressing *VrNHX1* constitutively (Lines 1–3, 35S::*VrNHX1*) and inducibly (Lines 4–6, RD29A::*VrNHX1*). NaCl-induced morphological changes was visible in 10 days old WT and transgenic lines after exposure to 200 mM NaCl for 5 days. (B) Changes in chlorophyll, MDA and proline content were estimated and analyzed as explained in materials and methods section. Values indicate means ± SE (n = 3). Statistically significant values at P≤0.05 are indicated as “*”, using Bonferroni analysis.

In order to characterize *VrNHX1* functionally *in planta*, T_3_ homozygous *Arabidopsis* lines expressing *VrNHX1* under the control of constitutive CaMV35S promoter or a stress-responsive RD29A promoter were generated using the binary constructs pCAMBIA2301-35S::*VrNHX1* (Fig. S5 A) and pCAMBIA2301-RD29A::*VrNHX1* (Fig. S5 B), respectively, to study their performance under salt stress. The germination efficiency was studied in transgenic lines 1 (35S::*VrNHX1*) and 4 (RD29A::*VrNHX1*) after exposure to 150 mM NaCl stress for 10 days. Under normal condition, no difference was observed in WT and transgenic lines (Fig. 9 A). However, the transgenic lines exhibited better survival and germination efficiency than WT under salt stress (Fig. 9 A). Further, inhibition of root growth in WT and transgenic lines under salt stress (150 mM NaCl) was studied (Fig. 9 B). Transgenic lines 1 and 4 exhibited 2.65 and 3 times higher root length respectively, than WT (Fig. 9 C). The effect on physiological parameters was monitored in 10 days old wild-type (WT) and independent transgenic *Arabidopsis* lines expressing *VrNHX1* constitutively (Lines 1–3, 35S::*VrNHX1*) and inducibly (Lines 4–6, RD29A::*VrNHX1*) under 200 mM NaCl stress for 5 days, by analyzing the total chlorophyll, malondialdehyde (MDA) for lipid peroxidation and proline content. Under normal physiological condition, no qualitative and statistical difference was observed between wild-type and transgenic *Arabidopsis* lines (Fig. 10). However, under salt stress (200 mM NaCl), WT showed leaf senescence while transgenic *Arabidopsis* lines (Lines 1–3, 35S::*VrNHX1* and Lines 4–6, RD29A::*VrNHX1*) showed better growth and survival (Fig. 10 A). The transgenic lines showed higher chlorophyll (18–20 mg/ml) and proline (4.8–6 µmoles/g FW) content than WT (Fig. 10 B). The 35S::*VrNHX1* lines showed 1.35 times higher proline than RD29A::*VrNHX1* lines. A lower lipid peroxidation was detected in transgenic lines as WT showed 1.33 times higher malondialdehyde (MDA) content (Fig. 10 B).

**Figure 11:**
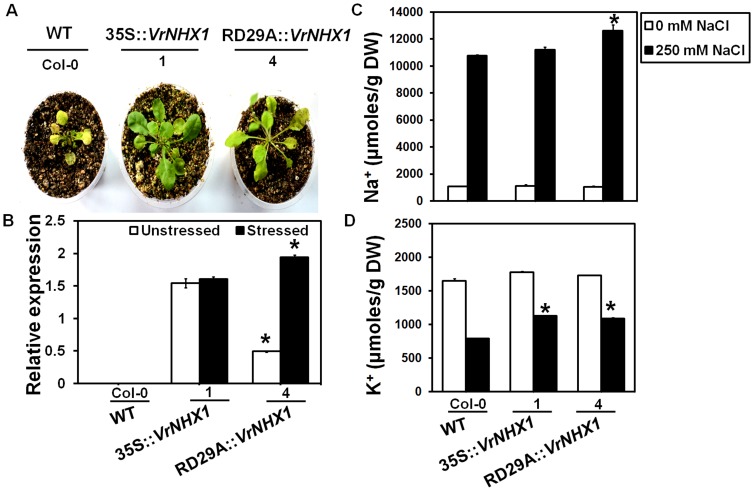
Salt tolerance assay in mature transgenic *Arabidopsis* lines under salt stress. (A) Effect of salt stress on wild type (WT, Col-0) and transgenic *Arabidopsis* lines expressing *VrNHX1* constitutively (Line 1, 35S::*VrNHX1*) and inducibly (Line 4, RD29A::*VrNHX1*) subjected to 250 mM NaCl treatment for 2 weeks (B) Relative transgene expression level of *VrNHX1* in transgenic *Arabidopsis* lines under unstressed and salt stressed conditions. No transgene expression was observed in WT. A 0.283 kb fragment of *VrNHX1*::35SployA and 0.150 kb fragment of *AtUBQ1* was amplified in quantitative RT-PCR analysis (C) Na^+^ and (D) K^+^ content (µmoles/g DW) was estimated in leaves of unstressed (0 mM NaCl) and salt stressed (250 mM NaCl) WT and transgenic lines, as described in materials and methods. Values indicate means ± SE (n = 3). Statistically significant values at P≤0.05 are indicated as “*”, using Bonferroni analysis.

Effect of salt stress was studied in mature WT and transgenic lines (Line 1, 35S::*VrNHX1* and Line 4, RD29A::*VrNHX1*). The transgenic lines displayed better survival efficiency while WT exhibited leaf senescence and growth inhibition upon salt stress (200 mM NaCl) (Fig. 11 A). Transgenic *Arabidopsis* 35S::*VrNHX1* plants displayed constitutively high expression of *VrNHX1* under both control (unstressed) and salt stress conditions, whereas RD29A::*VrNHX1* lines showed high induction of *VrNHX1* only after stress treatment with basal expression levels under normal conditions (Fig. 11 B). The total Na^+^ and K^+^ accumulated in transgenic lines was higher than WT. Further, transgenic 35S::*VrNHX1* and RD29A::*VrNHX1* lines exhibited 1.3 and 1.14 times higher Na^+^/K^+^ ratio, respectively, as compared to WT (Fig. 11 C, D).

## Discussion

This is the first report on isolation and functional characterization of a vacuolar Na^+^/H^+^ antiporter (*VrNHX1*) from mungbean. Phylogenetic analysis and evolutionary relationship revealed that *VrNHX1* shared highest homology with reported legume Na^+^/H^+^ antiporters belonging to the Class-I type NHX exchanger group. The potential structural and functional similarity between yeast and plant endosomal Na^+^/H^+^ exchanger, serves as a valuable tool for validation of novel plant Na^+^/H^+^ exchangers for their role in salt tolerance [35], [36]. Restored growth of AXTVrNHX1 cells in presence of high concentrations of Na^+^, K^+^, and Li^+^ and suppression of hygromycin sensitivity indicated the functional complementation of *ScNHX1* by heterologous expression of *VrNHX1*. The Na^+^ distribution pattern in vacuolar and cytoplasmic fractions of AXTVrNHX1 cells as compared to AXTYES2.0 cells, indicated the potent role of *VrNHX1* as a vacuolar Na^+^/H^+^ antiporter limited to vacuolar sequestration of alkali cations for establishing ion homeostasis. Similar findings were reported in functional complementation of *OsNHX1* in AXT3 mutant [37]. Moreover, *VrNHX1* expression in AXTVrNHX1 showed enhanced K^+^ distribution within vacuolar fractions which was in accordance with the results obtained in heterologous expression of *AtNHX1* [10] and *TNHXS1* [38] in AXT3 mutant. It was also observed that cytoplasmic K^+^ fractions were lower in AXTYES2.0 cells as compared with AXTVrNHX1 cells and W303-1B wild type cells. Alkalinization of endolytic compartments has been reported to be mediated by *ScNHX1* which serves as a leak pathway for H^+^, thus, regulating the pH level for efficient survival against external acid stress [27], [39]. In our studies, we observed that growth sensitivity of AXTVrNHX1 cells was lower than AXTYES2.0 cells under external acidic pH environment. Vacuolar acidification was reduced in AXTVrNHX1 cells under low pH indicating the role of *VrNHX1* in extrusion of excess H^+^ by its ion specificity.

Differential regulation of Na^+^ uptake, extrusion, compartmentalization, radial transport to stele, loading and unloading into xylem is responsible for the varied response of plants against salinity stress. Under salt stress, *VrNHX1* expression was induced in both leaves and roots of mungbean seedlings with concomitant higher expression in roots than leaves in both early and mid stage seedlings. This result was in accordance with previous reports on expression of *ZmNHX1*, *AeNHX1*, *AlNHX1*, and *ThNHX1* [40]–[43] and contrary to reports of expression *OsNHX1*, *AgNHX1*, *SsNHX1*, *PeNHX1*, *MsNHX1*, *TrNHX1*, *ZjNHX1*, *ZxNHX*, and *DmNHX1* [17], [44]–[51] which had higher expression in leaves/shoots. The expression pattern of *VrNHX1* under various abiotic stress conditions in mungbean revealed gradual increase in expression under salt stress (200 mM NaCl) after 24 hrs, cold stress (4°C) at 12 hrs and dehydration stress (200 mM mannitol) after 24 hrs. The result was contrary to the previous reports on expression pattern of *PeNHX1* and *ThNHX1* [42], [48] under cold stress that showed decrease in the transcript accumulation. No change in expression pattern of *AtNHX1* under cold stress has been reported [52]. Up-regulation of *VrNHX1* under cold stress can be attributed to the other unknown functional mechanisms that still remain to be deciphered. However, involvement of *NHX1* in conferring freezing tolerance has been reported in transgenic *A. thaliana* overexpressing *SsNHX1*, although the exact mechanism has not been explained [53]. Water deficit and altered water potential along with ionic imbalance are known to be primary effects of salt stress [4], [8]. We found under dehydration stress the expression pattern of *VrNHX1* in mungbean seedlings similar to previous reports on expression of *GmNHX1*, *ThNHX1* and *EgNHX1* which displayed up-regulation under dehydration stress [13], [42], [54]. However, contrasting results have been reported for expression of *PeNHX1* and *AtNHX1* [48], [52].

Physiological response under salt stress, indicated higher Na^+^ accumulation in roots than shoots in early and mid stage mungbean seedlings, in contrast to the reports in *T. repens*, *Z. japonica*, *H. caspica*, *Z. xanthoxylum*, *D. morifolium* [17], [49]–[51], [55] that showed preferential accumulation of Na^+^ in leaves/shoots. This indicated that higher K^+^/Na^+^ ratio is maintained in leaves owing to sequestration of higher Na^+^ in root vacuoles thus, restricting their movement to the aerial part of plant. Combined together, increased *VrNHX1* transcript level coupled with higher sequestration of Na^+^ in roots can be attributed as the tolerance mechanism of mungbean under salt stress.

Ectopic expression of *VrNHX1* conferred salt tolerance in transgenic *Arabidopsis* lines. Both, 35S::*VrNHX1* and RD29A::*VrNHX1* homozygous T_3_ lines displayed better growth response in comparison to WT. Salt stress affects the photosynthetic system components including chlorophyll contents [56]. The reduction in chlorophyll content was less in transgenic lines (35S::*VrNHX1* and RD29::*VrNHX1*) as compared to WT. Lipid peroxidation is mediated by increase in accumulation of reactive oxygen species (ROS) under salinity stress [57]. Therefore, the extent of lipid peroxidation was measured using malionaldehyde (MDA), a by-product of lipid peroxidation. Transgenic lines showed lower extent of MDA generation as compared to WT indicating protection against membrane damage process. Metabolic response against salt stress, generally includes generation of proline, an osmoprotectant and compatible osmolyte, as a protective measure in plants [4]. Transgenic lines expressed higher proline content in response to salt stress. Proline is also known as a potent ROS scavenger [58] which might also be correlated with the lower levels of generation of ROS, thus rendering reduced lipid peroxidation in transgenic plants as compared to WT. Similar result was also reported for proline content in transgenic *Arabidopsis* lines overexpressing *DmNHX1* [51]. The regulation of K^+^/Na^+^ ratio to maintain K^+^ homeostasis for proper cellular and enzymatic functioning is an essential mechanism against salinity stress in plants [59]. Our results demonstrated that the transgenic lines (35S::*VrNHX1* and RD29::*VrNHX1*) maintained a higher K^+^/Na^+^ ratio than WT plants under salt stress indicating effective tolerance in transgenic lines under salt stress. The phenotypical, physiological and expressional analysis using quantitative real-time PCR concluded that the transgenic RD29::*VrNHX1* line displayed comparable higher survival and growth than 35S::*VrNHX1* lines under salt stress and can be further exploited in crop plants.

The expression of *VrNHX1* under constitutive and inducible promoter enhanced salt tolerance in transgenic *Arabidopsis*. *AtNHX1* is one of the most effective genes in improving plant salt tolerance, however, it played a dominant role mainly in leaf. Our result suggested that *VrNHX1* might play an important role in the root resistance to Na^+^ toxicity. Therefore, we could assume that overexpression of *VrNHX1* in crop plants might generate enhanced salt tolerance.

## Supporting Information

Figure S1 Multiple sequence alignment was performed for amino acid sequences of plant NHX proteins using CLUSTAL W. The GenBank Accession numbers for NHX proteins are: VrNHX1 (AEO50758.1), *Vigna radiata*; VuNHX1 (AEO72079.2), *Vigna unguiculata*; GmNHX1 (AAY430061.1), G*lycine max*; CkNHX1 (ABG89337.1), *Caragana korshinskii*; MsNHX1 (AAS84487.1), *Medicago sativa*; CaNHX1 (ADL28385.1), *Cicer arietinum*; TrNHX1 (ABV00895.1), *Trifolium repens*; LtNHX1 (ACE78322.1), *Lotus tenuis*. “*” indicates identical amino acid (AA) residues. “:” indicates conservative AA substitutions and “.” represents semi-conservative AA substitutions in the sequence alignment. The transmembrane region of VrNHX1 as indicated by TM 1–11 and conserved amiloride binding motif, ^84^-LFFIYLLPPI-^93^, a classic inhibitor of the Na^+^/H^+^ antiporters detected in TM3 region is also shown in the alignment.
(TIF)

Figure S2 Prediction of transmembrane helices of VrNHX1 (AEO50758.1).The hydropathy plot was generated using TMPred online software. The positive values indicate putative transmembrane domains as indicated as TM 1–11.
(TIF)

Figure S3 Growth measurement of yeast strains under low pH. Yeast strains were grown in synthetic medium APGal (pH 4.0) and absorbance was measured at 600 nm. The data shown above are normalized to growth under normal condition (APGal, pH 7.0). W303-1B:- Wild type strain, AXTYES2.0:- AXT3 mutant harboring null pYES2.0 plasmid, AXTVrNHX1:- AXT3 mutant harboring pYES*VrNHX1* recombinant plasmid. Data represent mean from three independent events (n = 3) and standard error plotted in the graph. Statistically significant values at P≤0.05 are indicated as “*”, using Bonferroni analysis.
(TIF)

Figure S4 Calibration curve for pH sensitive BCECF fluorescent dye was plotted using standards ranging from pH 4.0–8.0. Yeast strains (W303-1B, AXTYES2.0, AXTVrNHX1) grown in APGal medium (pH 4.0) were loaded with BCECF dye as described in materials and methods, fluorescence intensity was measured at 440 and 490 nm, background values (measured with only cell extract and only BCECF dye) were subtracted and the ratio was plotted for each pH value. The data from the three yeast stains were pooled and mean ratio values were plotted with a fitted non-linear graph.
(TIF)

Figure S5 T-DNA region of pCAMBIA2301-35S::*VrNHX1* (13.9 kb) and pCAMBIA2301-RD29A::*VrNHX1* (14.4 kb). Restrcition enzyme PstI and EcoRI used for cloning 35SP::*VrNHX1*::35STer cassette (2.3 kb) and RD29A::*VrNHX1*::35STer cassette (2.8 kb) into plant binary vector pCAMBIA 2301 (11.6 kb) is also highlighted. Abbreviations: LB, left border; RB, right border; 35S Promoter, Cauliflower mosaic virus 35S promoter; RD29A promoter, Stress indicible AtRD29A promoter; CaMV 35S poly-A, Cauliflower mosaic virus 35S terminator; nos poly-A, nopaline transferase terminator; *nptII*, neomycin phosphotransferase; *intron-gus-A*, intron interrupted β-glucuronidase; *VrNHX1*, *Vigna radiata NHX1*.
(TIF)

Table S1 The putative post-translational modification sites predicted by ScanProsite software for VrNHX1.
(DOCX)
