# Supplementary figures and images for: Cloning and Functional Characterization of a Vacuolar Na+/H+ Antiporter Gene from Mungbean (VrNHX1) and Its Ectopic Expression Enhanced Salt Tolerance in Arabidopsis thaliana
Source: PLoS One. 2014 Oct 28;9(10):e106678. doi: 10.1371/journal.pone.0106678 (PMC4211658; doi:10.1371/journal.pone.0106678)

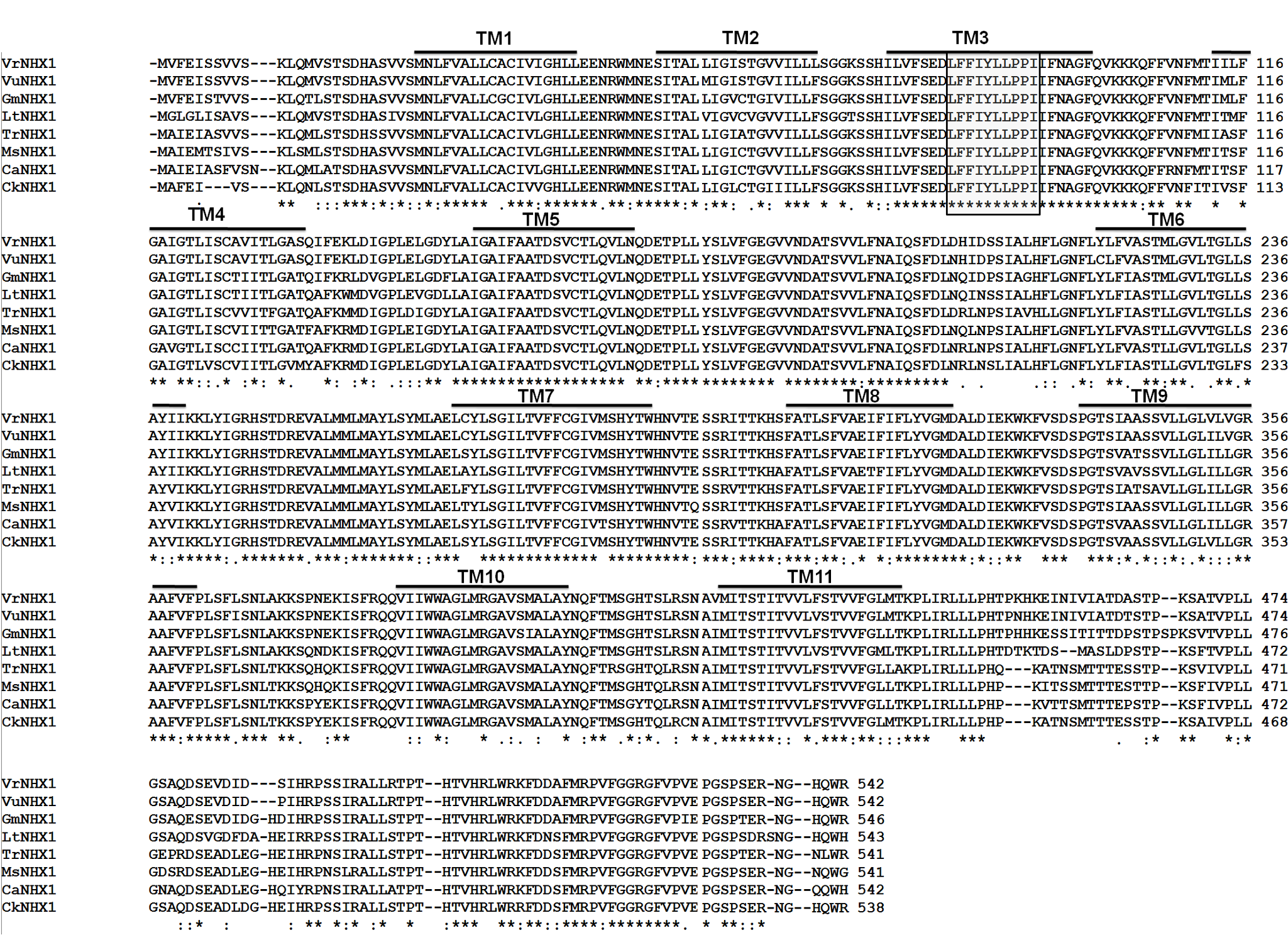

Supplement: Figure S1 — Multiple sequence alignment was performed for amino acid sequences of plant NHX proteins using CLUSTAL W. The GenBank Accession numbers for NHX proteins are: VrNHX1 (AEO50758.1), Vigna radiata; VuNHX1 (AEO72079.2), Vigna unguiculata; GmNHX1 (AAY430061.1), Glycine max; CkNHX1 (ABG89337.1), Caragana korshinskii; MsNHX1 (AAS84487.1), Medicago sativa; CaNHX1 (ADL28385.1), Cicer arietinum; TrNHX1 (ABV00895.1), Trifolium repens; LtNHX1 (ACE78322.1), Lotus tenuis. “*” indicates identical amino acid (AA) residues. “:” indicates conservative AA substitutions and “.” represents semi-conservative AA substitutions in the sequence alignment. The transmembrane region of VrNHX1 as indicated by TM 1–11 and conserved amiloride binding motif, 84-LFFIYLLPPI-93, a classic inhibitor of the Na+/H+ antiporters detected in TM3 region is also shown in the alignment. (TIF) [file pone.0106678.s001.tif]

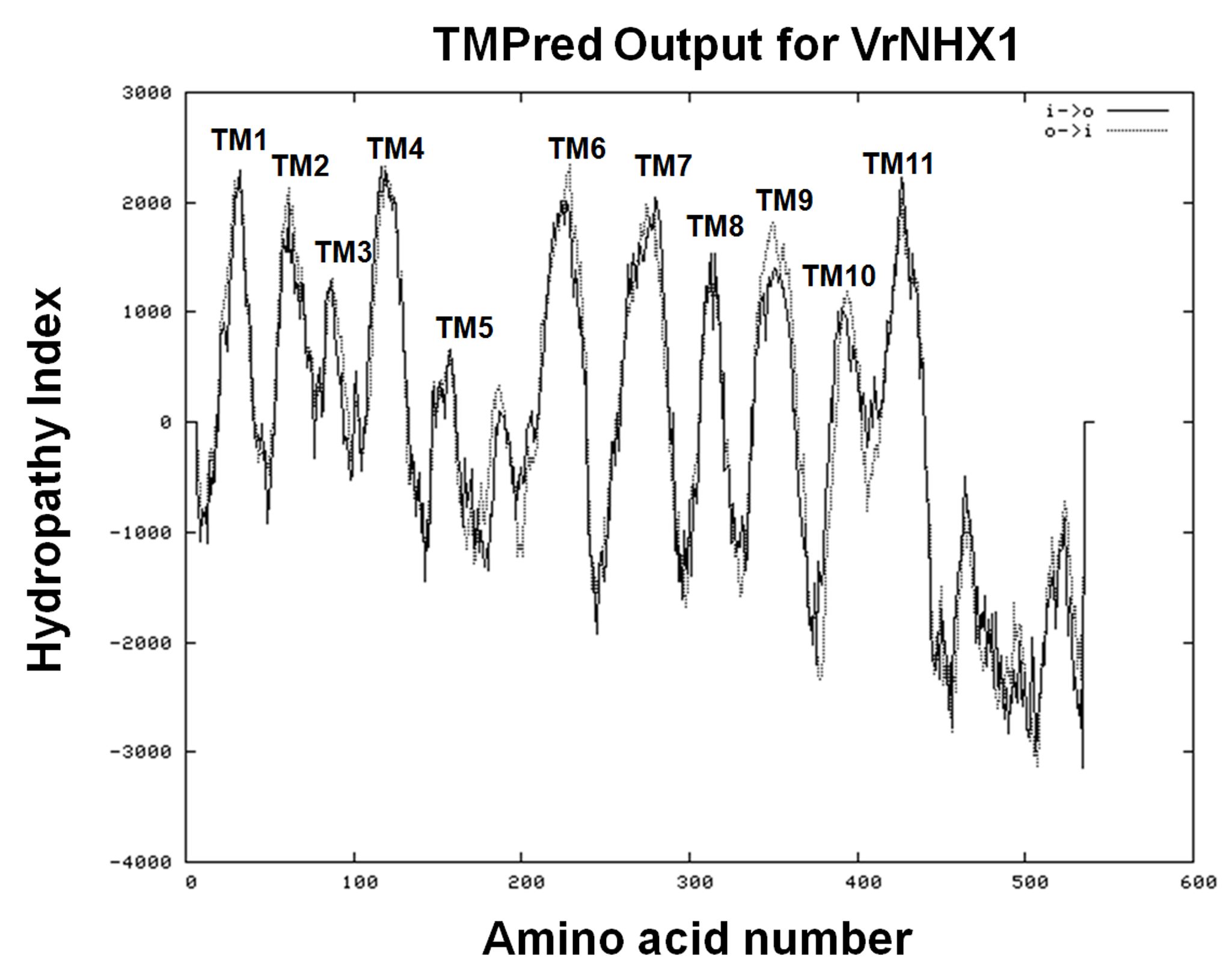

Supplement: Figure S2 — Prediction of transmembrane helices of VrNHX1 (AEO50758.1).The hydropathy plot was generated using TMPred online software. The positive values indicate putative transmembrane domains as indicated as TM 1–11. (TIF) [file pone.0106678.s002.tif]

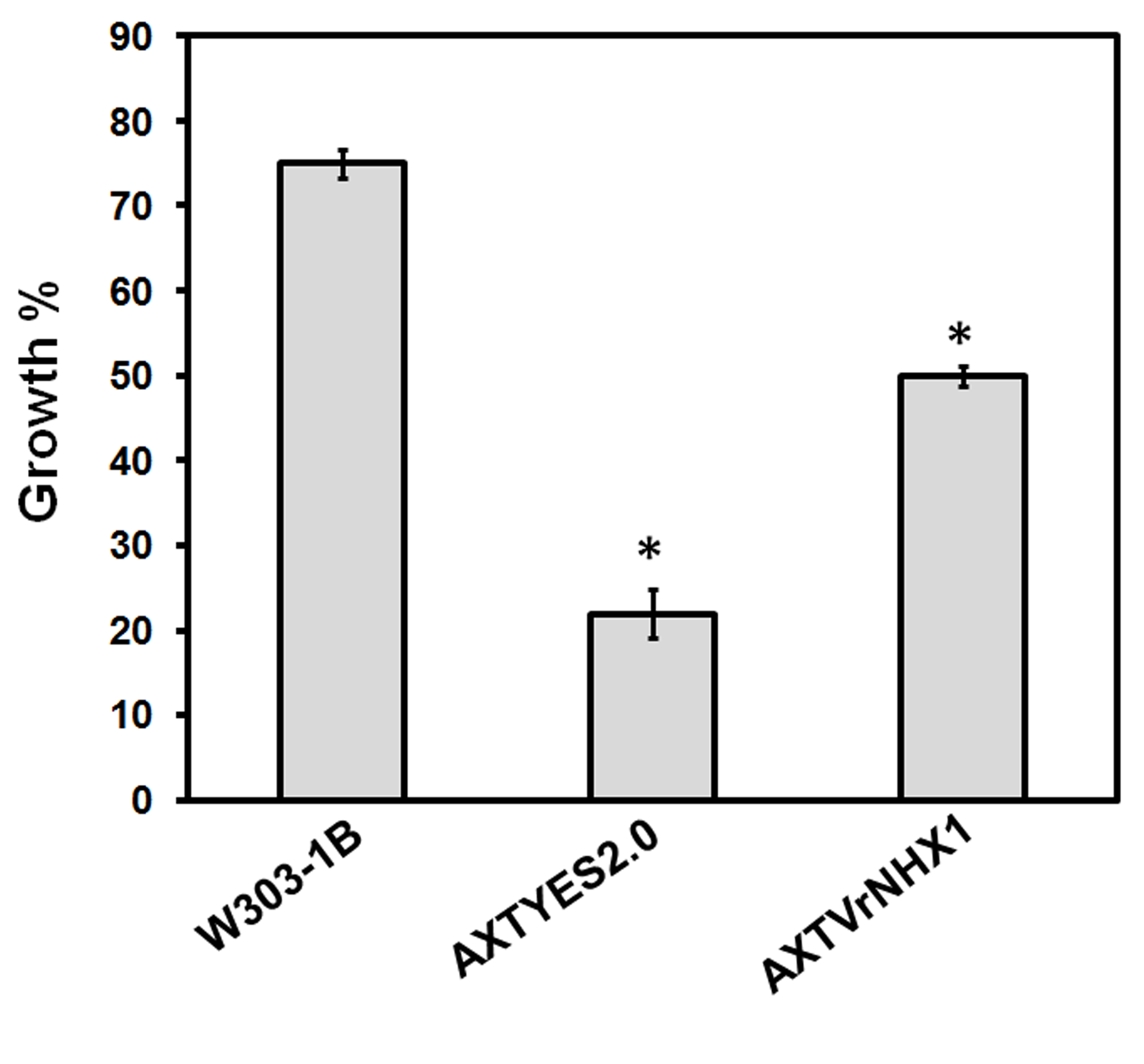

Supplement: Figure S3 — Growth measurement of yeast strains under low pH. Yeast strains were grown in synthetic medium APGal (pH 4.0) and absorbance was measured at 600 nm. The data shown above are normalized to growth under normal condition (APGal, pH 7.0). W303-1B:- Wild type strain, AXTYES2.0:- AXT3 mutant harboring null pYES2.0 plasmid, AXTVrNHX1:- AXT3 mutant harboring pYESVrNHX1 recombinant plasmid. Data represent mean from three independent events (n = 3) and standard error plotted in the graph. Statistically significant values at P≤0.05 are indicated as “*”, using Bonferroni analysis. (TIF) [file pone.0106678.s003.tif]

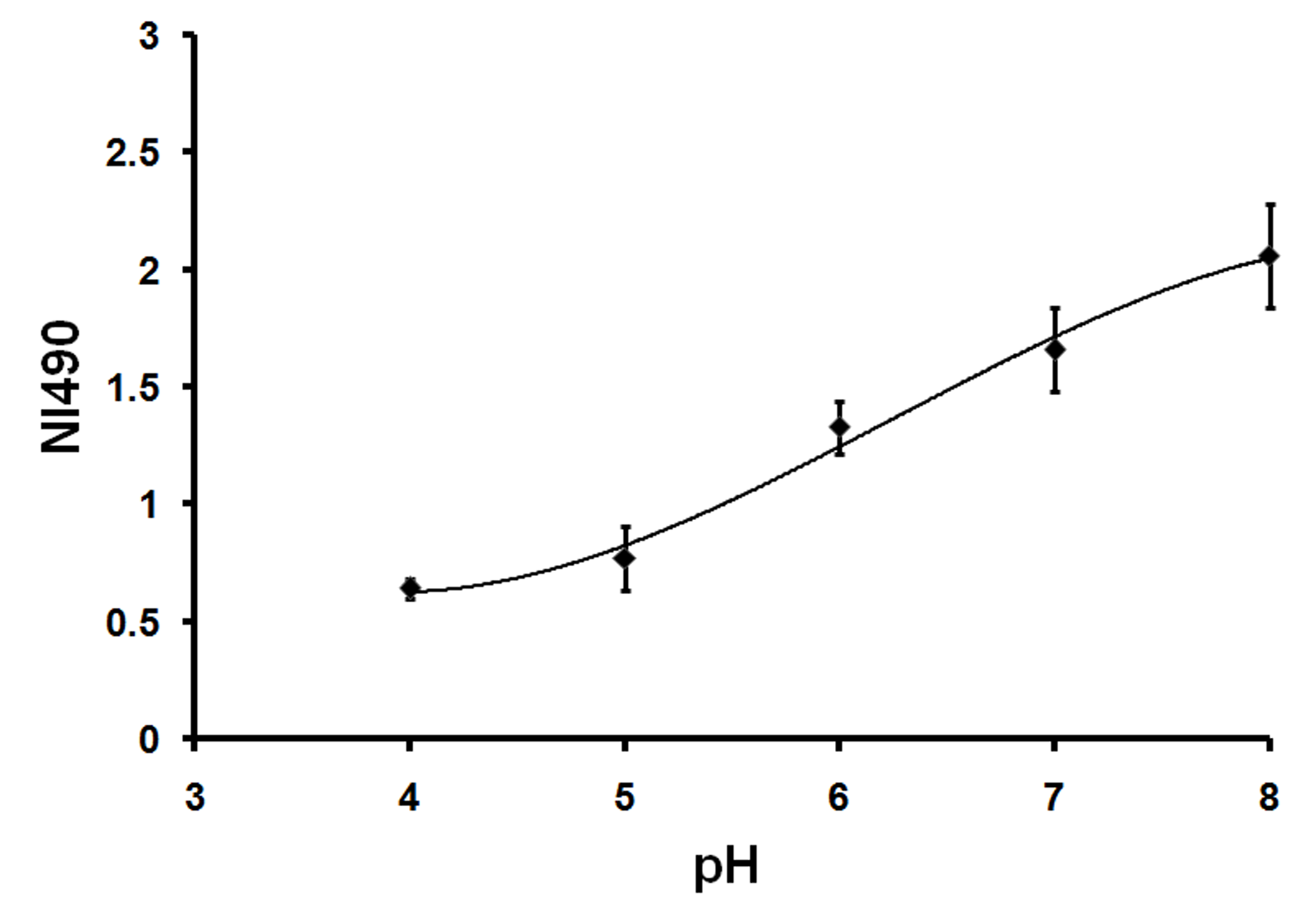

Supplement: Figure S4 — Calibration curve for pH sensitive BCECF fluorescent dye was plotted using standards ranging from pH 4.0–8.0. Yeast strains (W303-1B, AXTYES2.0, AXTVrNHX1) grown in APGal medium (pH 4.0) were loaded with BCECF dye as described in materials and methods, fluorescence intensity was measured at 440 and 490 nm, background values (measured with only cell extract and only BCECF dye) were subtracted and the ratio was plotted for each pH value. The data from the three yeast stains were pooled and mean ratio values were plotted with a fitted non-linear graph. (TIF) [file pone.0106678.s004.tif]

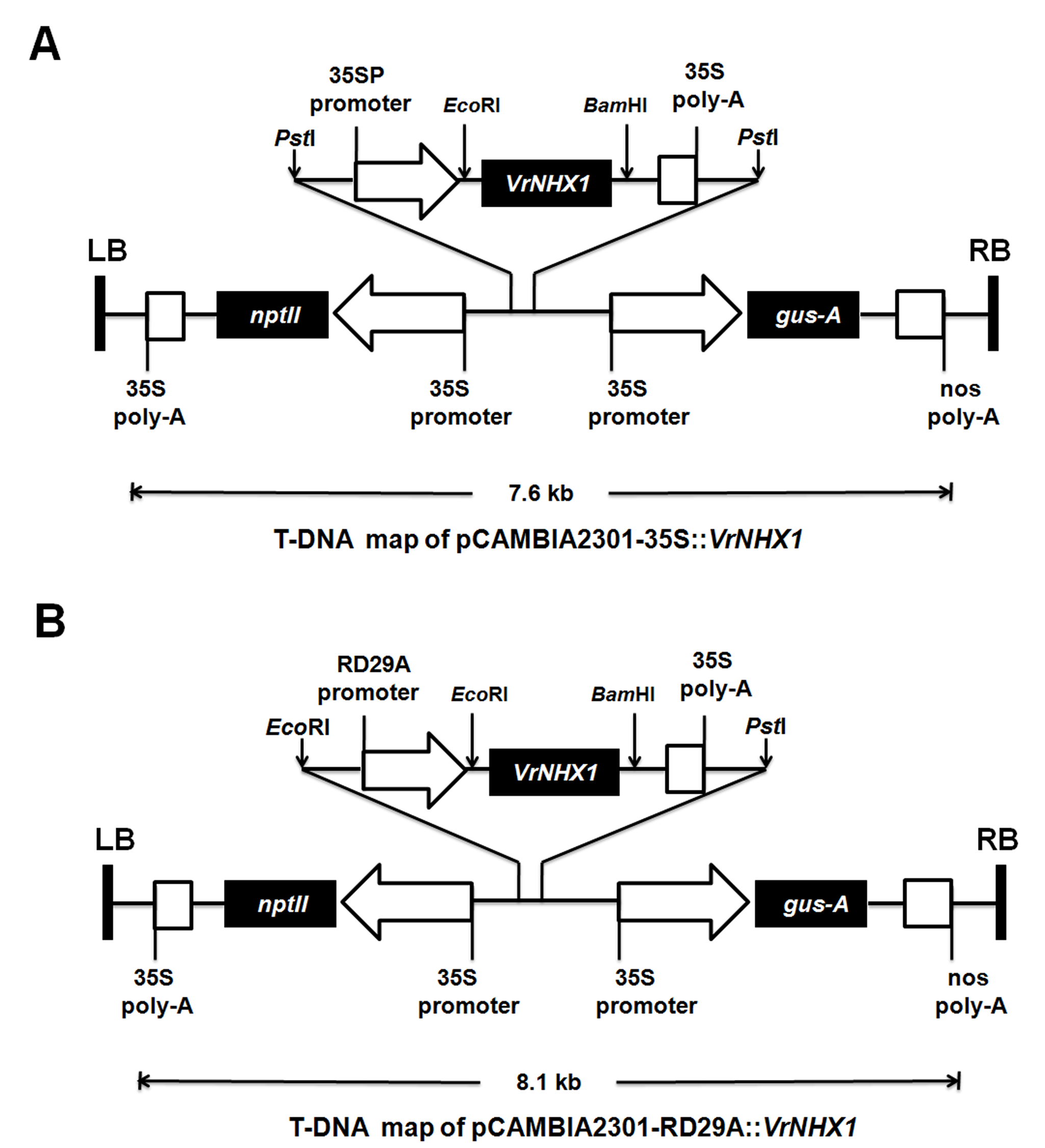

Supplement: Figure S5 — T-DNA region of pCAMBIA2301-35S::VrNHX1 (13.9 kb) and pCAMBIA2301-RD29A::VrNHX1 (14.4 kb). Restrcition enzyme PstI and EcoRI used for cloning 35SP::VrNHX1::35STer cassette (2.3 kb) and RD29A::VrNHX1::35STer cassette (2.8 kb) into plant binary vector pCAMBIA 2301 (11.6 kb) is also highlighted. Abbreviations: LB, left border; RB, right border; 35S Promoter, Cauliflower mosaic virus 35S promoter; RD29A promoter, Stress indicible AtRD29A promoter; CaMV 35S poly-A, Cauliflower mosaic virus 35S terminator; nos poly-A, nopaline transferase terminator; nptII, neomycin phosphotransferase; intron-gus-A, intron interrupted β-glucuronidase; VrNHX1, Vigna radiata NHX1. (TIF) [file pone.0106678.s005.tif]
