# Supplementary material for: Cloning and Functional Characterization of a Vacuolar Na+/H+ Antiporter Gene from Mungbean (VrNHX1) and Its Ectopic Expression Enhanced Salt Tolerance in Arabidopsis thaliana
Source: PLoS One. 2014 Oct 28;9(10):e106678. doi: 10.1371/journal.pone.0106678 (PMC4211658; doi:10.1371/journal.pone.0106678)
Supplement: Table S1 — The putative post-translational modification sites predicted by ScanProsite software for VrNHX1. (DOCX) [file pone.0106678.s006.docx]

**Table S1:** The putative post-translational modification sites predicted by ScanProsite software for VrNHX1

| **Predicted sites/patterns** | **Amino acid positions** |
| --- | --- |
| **ASN_GLYCOSYLATION** | 50-53  293-296 |
| **CK2_PHOSPHO_SITE** | 16-19  250-253  373-376  476-479  480-483  532-535 |
| **PKC_PHOSPHO_SITE** | 250-252  297-299  301-303  379-381  412-414  449-451  465-467  492-494  534-536 |
| **MYRISTYL** | 59-64  71-76  117-122  120-125  153-158  229-234  233-238  283-288  339-344  390-395 |
| **LEUCINE_ZIPPER** | 256-277 |

ASN_GLYCOSYLATION- N-Glycosylation site, CK2_PHOSPHO_SITE- Casein kinase II phosphorylation site, PKC_PHOSPHO_SITE- Protein kinase C phosphorylation site, MYRISTYL- N-Myristylation site, LEUCINE_ZIPPER- Leucine zipper pattern
